# Improvement of mobility and motivation in patients with elective colorectal resection using tracking devices and utilizing self-awareness (IMPETUS): a randomized controlled trial in a traditional non-ERAS clinical setting

**DOI:** 10.1186/s12893-026-03864-6

**Published:** 2026-05-27

**Authors:** L. Zimniak, E. Soufiah, C. S. R Ha, S. Gretschel, M. Paschold

**Affiliations:** 1 Department of General, Visceral, Thoracic and Vascular Surgery, University Hospital Ruppin- Brandenburg Neuruppin, Fehrbelliner Straße 38, Neuruppin, 16816 Germany; 2 Faculty of Health Sciences Brandenburg, Brandenburg Medical School Theodor Fontane, Brandenburg, Germany; 3 Department of General and Visceral Surgery, St. Marienwörth Hospital, Academic Teaching Hospital of the University Medical Center Mainz, Bad Kreuznach, Germany; 4 Department of General and Visceral Surgery, Kreisklinik Groß-Umstadt, Kreiskliniken Darmstadt- Dieburg, Groß-Umstadt, Germany; 5 Institute for Medical Biostatistics, Epidemiology and Informatics (IMBEI), University Medical Centre of the Johannes Gutenberg-University, Mainz, Germany

**Keywords:** Activity tracking, Enhanced recovery after surgery, Smart wearables, Patient motivation, Postoperative mobility

## Abstract

**Background:**

Early postoperative mobilization is a cornerstone of modern perioperative care, including in elective colorectal surgery. Here we evaluated the effectiveness of smart wearables for improving patients’ mobility and motivation during the recovery period after elective colorectal resection.

**Methods:**

This prospective randomized two-armed clinical trial enrolled 62 patients undergoing elective colorectal resection. These patients were randomized into an intervention group that received hourly active reminders to mobilize via a smartwatch, and a control group that received only passive monitoring. All patients were given a wearable device that tracked their daily step counts. The primary end-point was the total number of steps taken within five postoperative days. Secondary end-points included complication rates, length of hospital stay, and subjective motivation, assessed using a structured questionnaire.

**Results:**

Compared to the control group, the intervention group achieved significantly higher step counts (*p* = 0.039), and reported increased motivation for mobilization (*p* = 0.016). The groups did not significantly differ in overall complication rates or duration of hospital stay.

**Conclusion:**

Smart wearable devices with hourly active reminder functionalities effectively promoted postoperative mobility and motivation.

**Trial registration:**

German Clinical Trials Register (DRKS), DRKS00039731. Registered 24.03.2026. Retrospectively registered.

**Supplementary Information:**

The online version contains supplementary material available at 10.1186/s12893-026-03864-6.

## Introduction

Perioperative treatment for major visceral surgery has changed over the past twenty years, with the testing and implementation of various methods—including the fast-track pathway and enhanced recovery after surgery (ERAS) procedure. ERAS is a clinical strategy that reduces postoperative stress and expedites recovery through a range of preoperative, intraoperative, and postoperative interventions [1]. Notably, early mobilization is a key component of postoperative recovery and has been associated with fewer complications after major elective surgery and improved patient care outcomes following abdominal procedures [2, 3]. The implementation of a structured ERAS program is also associated with reduced hospital stay durations especially in elective colorectal surgery [4–10].

Despite the demonstrated importance, little information is presently available regarding the influence of various methods of monitoring and increasing postoperative mobilization. The introduction of smart wearable technology for self-tracking—including activity trackers—represents an advancement in our ability to automatically measure human mobility. These smart devices are easy to use, commercially available, and capable of supporting mobility through reminder functions [11].

Previous studies suggest that different strategies to promote postoperative mobilization may have varying effectiveness. Passive approaches, such as activity monitoring alone or providing feedback on step counts, have shown inconsistent results [12]. In contrast, active interventions that directly prompt or encourage patients to mobilize—such as structured reminders or guided activity programs—may have a stronger impact on behavioral change. However, it remains unclear to what extent increases in step counts represent clinically meaningful improvements, particularly in the early postoperative period. Therefore, we conducted the IMPETUS trial to evaluate whether an hourly wearable-based reminder system improves postoperative mobility and motivation compared with passive monitoring alone in patients undergoing elective colorectal resection. We hypothesized that active reminders would increase patients’ early postoperative ambulation. The primary endpoint was cumulative step count during the first five postoperative days, a time window in which mobilization typically transitions from assisted to increasingly independent activity and in which early differences in ambulation are most likely to be attributable to the intervention rather than later recovery variability. Secondary endpoints included postoperative complications, length of hospital stay, and patient-reported motivation assessed by questionnaire.

## Materials and methods

We designed a prospective two-armed randomized clinical trial, with the aim of investigating the impact of a structured motivational alarm system using smart wearables in the postoperative setting. The study population included consecutive patients with both benign and malignant disease as well as different types of colorectal resections. Recruitment took place from October 2023 to April 2024, and follow-up ended at hospital discharge. While in the recovery room after surgery, all patients received the smart wearable, a Samsung Galaxy Watch Active 2^®^ in a 44-mm graphite casing. This device was able to monitor physiological parameters and activity metrics, including daily step count. The smart watch recorded step counts by real-time measuring, and was connected via Bluetooth to a smartphone (Xiaomi Redmi Note 3^®^), which collected the measured parameters using a health application (Samsung Health^®^). All patients had access to their daily performed step counts.

Participants were allocated using simple 1:1 randomization without blocking or stratification. The randomization sequence was generated by an independent investigator not involved in recruitment or clinical care using IBM SPSS Statistics 23 (IBM, Armonk, NY, USA). Allocation concealment was ensured by using sequentially numbered, opaque, sealed envelopes. After written informed consent and baseline assessment, the recruiting investigator enrolled participants and assigned them to the next available allocation according to the concealment procedure. The randomization list was stored securely and was not accessible to the recruiting clinicians.

All patients were treated according to the same postoperative clinical protocol. Perioperative management followed the institution’s standard clinical pathway. Our center is not a formal ERAS hospital and therefore does not implement a comprehensive ERAS bundle. All patients received standardized perioperative antibiotic prophylaxis and venous thromboembolism prophylaxis according to local guidelines. Postoperative nausea and vomiting prophylaxis and multimodal analgesia were administered per routine practice, with escalation as clinically indicated. Oral intake was resumed as tolerated, and progression to a soft diet was guided by clinical tolerance. Surgical drains and urinary/central venous catheters were managed according to clinical indication and removed at the discretion of the treating team. Discharge was based on routine clinical criteria, including clinical stability, adequate oral intake, sufficient pain control with oral analgesics, and adequate mobilization. Postoperative mobilization was encouraged in all patients as part of routine ward care. The interventional group received an active prompt to mobilize every hour between 8 am and 6 pm, via an alarm on the smart watch. The alarm signal included both an acoustic and a vibration signal (active stimulation). The control group was only monitored and could see their steps taken (passive stimulation). Due to the nature of the intervention (auditory/vibration reminders), participant blinding was not feasible. Clinical staff were not explicitly informed of group allocation beyond routine device handling. Outcome assessment for the primary endpoint (step counts) was based on device-recorded data; data export and preprocessing were performed using predefined procedures. All participants wore their smart watch day and night. The nursing staff removed the watch to charge the battery for one hour every night, while the patients were sleeping.

The sample size calculation was based on an expected moderate effect size (Cohen’s d = 0.63), with an alpha level of 0.05 and a statistical power of 0.8. As high-quality data specifically addressing wearable-based interventions in colorectal surgery are limited, this estimate was informed by the available literature, including the study by Wolk et al., and should be interpreted as an approximation [13].

We analyzed the cumulative steps taken after five postoperative days, and clinical outcome parameters—including length of hospital stay, time until starting a soft diet, total pain levels and others. Length of stay (LoS) was defined as the number of days from the day of surgery to the day of hospital discharge. Patients were discharged according to routine clinical criteria, including clinical stability, adequate oral intake, mobilization as tolerated, and pain control with oral analgesics. Total pain was defined as the patient-level median of daily numeric rating scale (NRS, 0–10) pain scores recorded once per day over postoperative days 0–5 (0 = no pain, 10 = worst pain). Time to start a soft diet was defined as the postoperative day on which the patient first tolerated solid/soft food without clinically relevant nausea or vomiting, based on routine ward documentation.

Postoperative complications were recorded and graded according to the Clavien–Dindo classification during the in-hospital period until discharge. The modified Charlson Comorbidity Index (mCCI, excluding age) was calculated from comorbidities documented in the medical records at admission using the Charlson weighting scheme. The patients completed a structured questionnaire before they were released from the hospital. Data analysis was performed using IBM SPSS Statistics 23 (IBM, Armonk, NY, USA). The Mann-Whitney U test and Fischer´s exact test were used to determine significance. All comparisons were considered significant at a level of *p* < 0.05.

We prespecified exploratory subgroup analyses using negative binomial regression models with interaction terms for surgical approach (laparoscopic vs. open). Models were adjusted for age, sex, BMI, and smoking status. Incidence rate ratios (IRR) with 95% confidence intervals (CI) were reported, and interaction p-values were derived from Wald tests.

Approval was obtained from the responsible ethics committee (Rhineland-Palatinate State Medical Association – Ethics Committee, application number 2021–15734), and the study was accredited by the German Cancer Society (ST-D500). Conduct and reporting followed the CONSORT guidelines, and all procedures were performed in accordance with the Declaration of Helsinki. The trial was retrospectively registered in the German Clinical Trials Register (DRKS00039731).

## Results

**Fig. 1:**
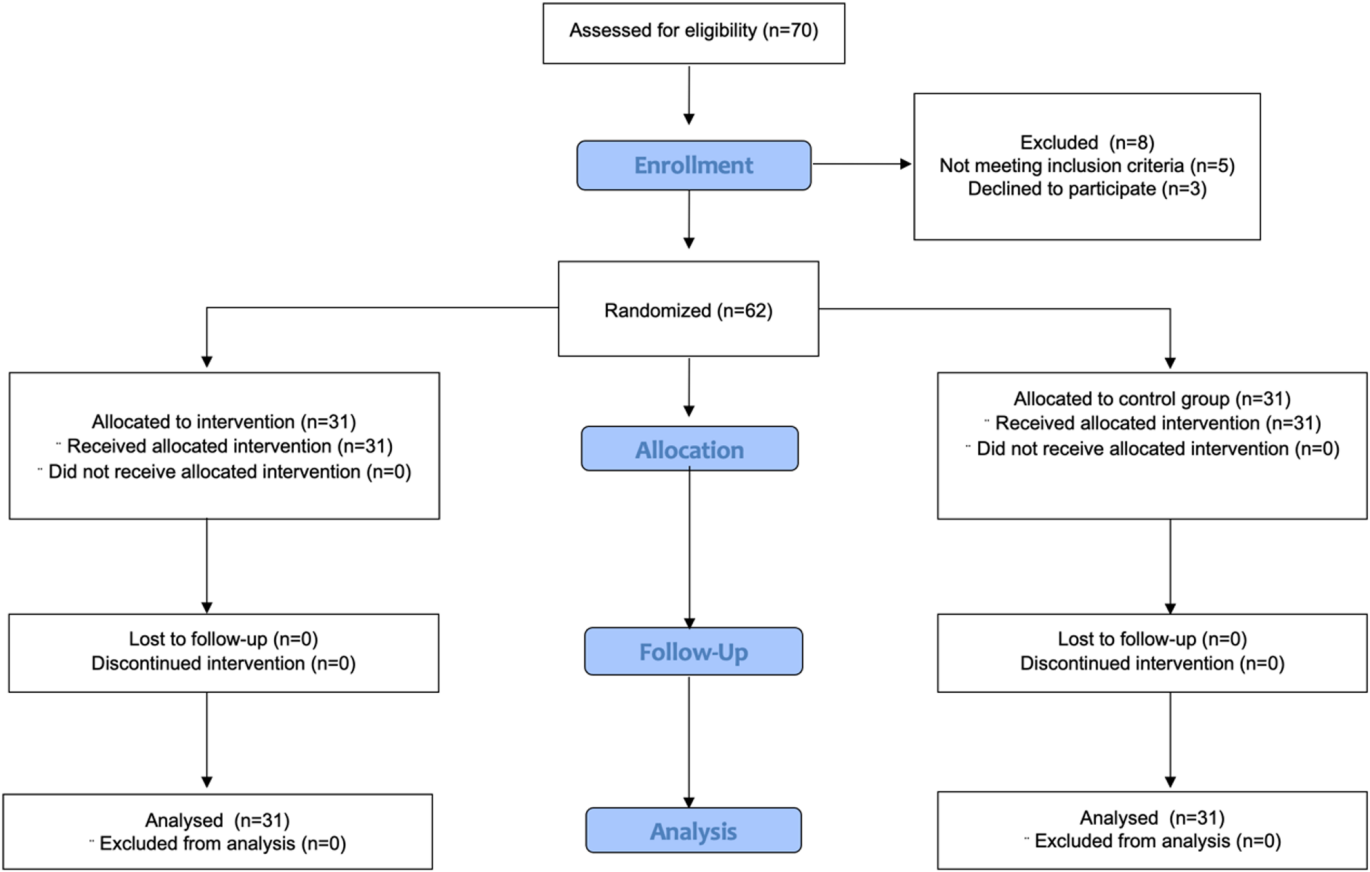
CONSORT flow diagram of patient enrollment, randomization, allocation, follow-up, and analysis

**Fig. 2:**
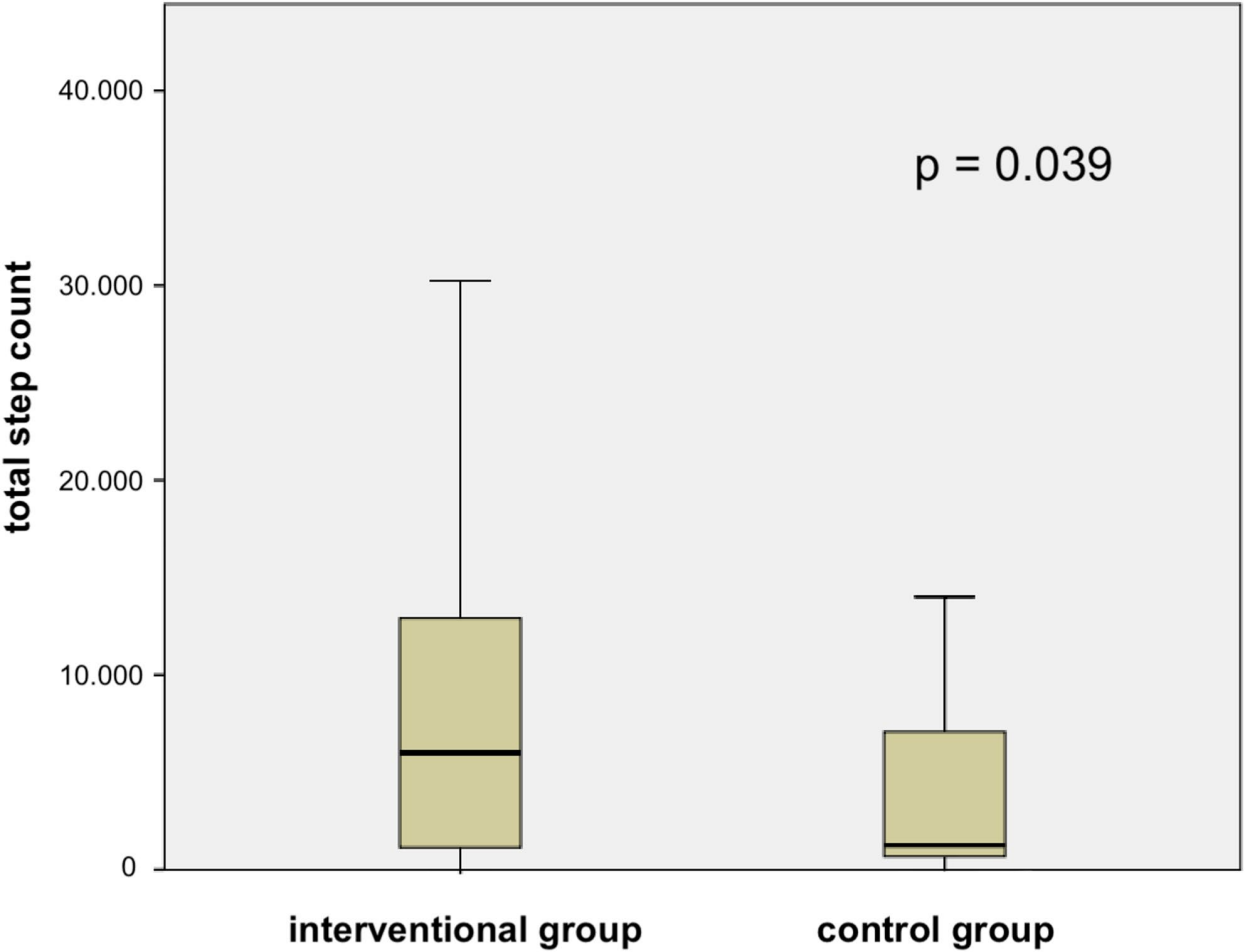
Statistical difference between the interventional and control groups, regarding total step counts within five postoperative days

A total of 70 patients were assessed for eligibility and 62 were randomized to the intervention group (*n* = 31) or control group (*n* = 31). All participants received the allocated intervention. Losses to follow-up and exclusions from analysis are shown in Fig. 1. The data set including 38 males and 24 females. The control group included a higher proportion of male patients compared to the intervention group; however, this difference was not statistically significant (*p* = 0.192). A detailed overview of patient screening, randomization, and inclusion in the final analysis is provided in (Fig. 1). No statistically significant differences were observed between groups in baseline characteristics, including age, sex, BMI, and surgical indication (Table 1). Among the 62 patients, 43 had malignant tumors, and 19 had benign diseases. A laparoscopic surgical approach was used in 87% of procedures. The median operation time in the whole cohort was 147 min (IQR = 73 min). The ASA score was 2 or 3 in 96.6% of patients, and 4 in 3.4% of patients. The surgical procedure was left hemicolectomy with anastomosis in 48.4%, right hemicolectomy with anastomosis in 38.7%, and rectal anterior resection with anastomosis in 12.9% of patients. A drainage system was placed in 91.9% of surgeries, and a central venous catheter (CVC) was placed in 77.4%. Step-count data were available for all participants in the intervention group and in the control group. Among the entire patient population, the median number of steps taken was 2856 (IQR = 11,528) within 5 days postoperatively. The intervention group took significantly more total steps than the control group (*p* = 0.039) (Fig. 2). Daily step counts per postoperative day (POD 0–5) are provided in Supplementary Table S1. Both groups showed a progressive increase in postoperative mobility over time. The intervention group consistently demonstrated higher step counts across all postoperative days. Statistically significant differences were observed on POD 1 (*p* = 0.013) and POD 4 (*p* = 0.033). Various clinical outcomes were compared between the interventional group and control group (Table 2). The other measured parameters did not significantly differ between the two groups. Analysis of the structured postoperative questionnaire, which patients completed using a 5-point Likert scale, revealed that patients in the interventional group experienced the subjective feeling of being more motivated to be active due to using the smart wearable (*p* = 0.016) (Table S2). 

**Table 1:** Baseline characteristics of the study population presented as n (%) or median [IQR]

Item	Interventional group *n* / median (IQR)	Control group *n* / median (IQR)	Overall *n* / median (IQR)
Age in years	67.0 [53.0–75.0]	66.0 [61.0–81.5]	66.5 [56.0–78.5]
Male	16 (51.6%)	22 (71.0%)	38 (61.3%)
Female	15 (48.4%)	9 (29.0%)	24 (38.7%)
BMI in kg/m²	25.0 [24.5–28.0]	27.0 [24.5–29.0]	26.0 [24.2–29.0]
Modified Charlson Comorbidity Index	2 [1–3]	2 [2–3]	2 [1–3]
ASA: ASA II	15 (48.4%)	15 (48.4%)	30 (48.4%)
ASA: ASA III	16 (51.6%)	14 (45.2%)	30 (48.4%)
ASA: ASA IV	0 (0.0%)	2 (6.5%)	2 (3.2%)
Malignant	21 (67.7%)	22 (71.0%)	43 (69.4%)
Benign	10 (32.3%)	9 (29.0%)	19 (30.6%)
Laparoscopic approach	27 (87.1%)	27 (87.1%)	54 (87.1%)
Open approach	4 (12.9%)	4 (12.9%)	8 (12.9%)
Smoker	13 (41.9%)	13 (41.9%)	26 (41.9%)

**Table 2:** Postoperative course and patient-reported outcomes in the intervention and control groups (median [IQR]); *P* values from between-group comparisons

Item	Interventional group *n* / median (IQR)	Control group *n* / median (IQR)	*P* value
Total steps (POD 0–5)*	6001.0 [1136.0–12917.0]	1251.0 [677.0–7081.5]	**0.039**
Length of hospital stay (days)	11.0 [9.0–17.5]	13.0 [9.0–20.0]	0.979
Drain removal time (days)	4.0 [3.0–5.0]	4.0 [3.0–5.0]	0.407
Foley catheter removal (days)	2.0 [1.0–3.0]	2.0 [1.0–3.5]	0.280
CVC removal (days)	2.0 [0.2–4.0]	3.0 [1.5–4.0]	0.454
Duration of operation (min)	140.0 [109.5–176.5]	154.0 [112.0–174.0]	0.900
Total pain (NRS 0–10)**	8.0 [6.5–9.5]	8.0 [6.0–9.5]	0.296
Time to start oral painkillers (days)	2.0 [1.0–3.0]	2.0 [2.0–3.8]	0.695
Time to start soft diet (days)***	4.0 [3.0–4.0]	4.0 [4.0–5.0]	0.232
Any complication (Clavien–Dindo I–V)	10 (32.3%)	13 (41.9%)	0.600
Clavien–Dindo grade I–II	8 (25.8%)	8 (25.8%)	1.000
Clavien–Dindo grade III–V	2 (6.5%)	5 (16.1%)	0.424

*Cumulative step count from postoperative day 0 through day 5 recorded by the wearable device

**Patient-level median of daily NRS (0–10) pain scores recorded once per day over POD 0–5

***Postoperative day on which a soft diet was first tolerated without clinically relevant nausea or vomiting

In the exploratory subgroup analysis, the intervention effect appeared to differ by surgical approach. In patients undergoing laparoscopic surgery, the intervention was associated with increased postoperative step counts (IRR 2.83, 95% CI 1.63–4.89). In contrast, estimates in patients undergoing open surgery suggested no consistent beneficial effect (IRR 0.17, 95% CI 0.04–0.75). The interaction between intervention and surgical approach was statistically significant (*p* < 0.001). Analyses showed no statistically significant association between postoperative step counts and sex, age (< 65 vs. ≥ 65 years), or major complications (Clavien–Dindo > II), although interpretation is limited by sample size.

## Discussion

Early postoperative mobility is a central element of modern perioperative treatment concepts, and promoting sufficiently high mobility can contribute to improving outcomes. One method to encourage mobility is through the use of smart wearables; however, scarce data about these tools are available in the current literature (Table 3). Studies have investigated the use of active or passive stimuli to improve postoperative mobility. Passive incentives include allowing patients to control their daily activity [13, 14], and giving patients rewards if they achieve an agreed-upon goal [15]. Active stimuli include external positive reinforcement of mobility motivation, such as requests from hospital staff [16] or motivational interviewing [17]. 

**Table 3:** Studies evaluating postoperative mobilization by active and passive stimulation

Study	Year	*N*	Methods and study design	Intervention	Stimulus type	Primary end-point	Results with statistical significance
Wolk et al. [13]	2019	132	Block randomization between laparoscopic and open surgery	Intervention group had feedback on daily step amount	passive	Average step count during the first five postoperative days	Increased step count in the laparoscopic group; lower hospital stay and lower morbidity among patients with more than the average step count
Reed et al. [14]	2023	235	Randomization of into two groups after bariatric surgery	Intervention group was informed about their daily step amount	passive	Number of steps during hospital stay post-surgery	No significant differences in step count between groups; lower step counts associated with increased age and prolonged hospital stay
Strother et al. [15]	2021	34	Randomization into two groups after cystectomy	Daily step goals were set; patients received financial reward if step goals were achieved	passive	Number of days postoperative step goals were met	No significant differences in primary outcomes between the groups
Niet al. [16]	2018	120	Randomization into two groups after liver resection	Postoperative activity program specified by the treating nurse	active	Postoperative step count, pain levels, sleeping time, and time to first postoperative bowel movement	Higher step count in interventional group; quicker restoration of bowel function and shorter hospital stay in early mobilization group
Wiesenberger et al. [17]	2024	60	Randomized two-armed patient-blinded pilot study	Motivational interviewing (MI)	active	Time out of bed (postoperative days 1–3)	Interventional group (IG) achieved longer time out of bed

Analyses of the studies that have used only passive stimulation show heterogeneous results. In the studies by Wolk et al. and Reed et al., patients in the interventional group had access to their step count [13, 14]. In a study by Strother et al., daily step goals were set, and patients who achieved these goals were rewarded [15]. The study by Wolk et al. describes a study protocol rather than a completed clinical trial, and therefore provides only limited information on clinical outcomes. In this context, reported findings suggesting an improvement in cumulative step counts, particularly in patients undergoing laparoscopic surgery, should be interpreted with caution. The authors hypothesized that differences between surgical approaches may be related to varying levels of intraoperative stress. Two previous studies have tested active stimulation in different patient populations, and both have shown higher step levels in their interventional groups. In the study by Ni et al., liver resection patients had to complete differently structured activity programs that were specified by the treating nurse. They observed quicker restoration of bowel function and a shorter hospital stay in their interventional group [16]. In the other study, Wiesenberger et al. [17] applied active stimulation in patients with elective colorectal surgery. Motivational interviewing was performed and postoperative mobility was monitored using step counters, resulting in a higher step count in the interventional group. Compared with previous studies using passive monitoring alone, our intervention combining passive tracking with active reminder prompts resulted in a clearer increase in postoperative step counts. In contrast to motivational interviewing approaches, the present intervention required no additional personnel resources and was fully automated, which may facilitate implementation in routine clinical practice. However, this increase in physical activity did not translate into measurable differences in clinical outcomes such as length of hospital stay or other postoperative recovery parameters. This lack of effect is not unexpected, given that clinical recovery is influenced by multiple perioperative factors and early mobilization represents only one component of a broader ERAS framework. In the absence of a fully implemented ERAS program, improvements in a single behavioral parameter—such as mobility—are unlikely to produce detectable changes in complex clinical endpoints. Our findings therefore demonstrate that while wearable-based reminders effectively enhance postoperative mobilization, they do not, on their own, appear sufficient to modify short-term clinical outcomes in this setting.

However, the use of these devices is generally not perceived as disruptive or unpleasant. The majority of our patients reported that the wearable device was not disturbing or difficult to use, and that they were able to perceive the mobility signal very well. No technical problems were experienced while using the system. Moreover, evaluation of the structured questionnaire revealed that the personal feeling of motivation to be active was significantly correlated with the actual number of steps taken. These responses and the statistical analysis support the conclusion that patients were motivated to be more active by using smart wearables with a mobility reminder alarm, without impairing their comfort or satisfaction with treatment. Rosenberg et al. [18] also reported that patients agreed that smart wearables are useful.

A key aspect when interpreting our findings is the perioperative setting. In contrast to fully implemented ERAS pathways, our center follows a more traditional protocol, reflected by longer hospital stays and delayed mobilization. Consequently, baseline postoperative mobility in our cohort was lower than that reported in ERAS-based studies. Within this context, wearable-based reminder interventions may be particularly effective in settings with low baseline mobilization, where structured prompts can help overcome early barriers to activity. However, generalizability to optimized ERAS pathways may be limited, as the additional benefit of such interventions could be less pronounced in already highly standardized care environments. Importantly, our results reflect real-world clinical practice in many institutions where full ERAS implementation has not yet been achieved. Compared to ERAS-based cohorts reporting substantially higher early postoperative step counts, the absolute mobility levels observed in our study were markedly lower, highlighting the influence of perioperative care structures on postoperative activity.

In an exploratory subgroup analysis, the intervention effect appeared to differ by surgical approach. Patients undergoing laparoscopic surgery showed a more pronounced increase in postoperative step counts compared to those treated with an open approach. This may reflect differences in surgical trauma and recovery dynamics, as minimally invasive procedures are generally associated with reduced pain and earlier mobilization. In contrast, no consistent beneficial effect was observed in the open surgery subgroup, suggesting that under conditions of higher physiological stress, behavioral interventions alone may be insufficient to substantially influence early postoperative activity. These findings should be interpreted with caution due to the limited sample size and exploratory nature of the analysis.

This study has several limitations. First, although the sample size was powered to detect differences in postoperative step counts, it was insufficient to identify changes in clinical outcomes, limiting conclusions regarding complications or length of stay.Second, the heterogeneous patient cohort—including both benign and malignant indications, different colorectal procedures may be associated with varying degrees of postoperative impairment. For example, rectal resections may lead to greater postoperative limitations compared with hemicolectomies, potentially influencing mobilization patterns independent of the intervention.

Third, the single-center setting and the absence of a fully implemented ERAS program may restrict generalizability to institutions with more standardized pathways. Moreover, although step counts were objectively recorded, other relevant mobility parameters such as time out of bed or gait speed were not assessed. Finally, individual patient factors such as baseline functional status or motivation were not controlled for and may have influenced responsiveness to reminder-based activation. Larger multicenter trials with more standardized pathways and comprehensive mobility metrics are needed to determine whether increased postoperative activity translates into clinical benefit. A further limitation relates to the accuracy of wrist-worn activity trackers. Previous studies have reported measurement deviations of up to 10%, particularly in settings with slow or irregular gait patterns. This is of particular relevance in the postoperative period, where patients often exhibit reduced walking speed and altered movement dynamics. This observation is in line with previous findings reported by Wiesenberger et al. [19] In addition, discrepancies between wrist-worn devices and validated motion sensors have been described, potentially leading to under- or overestimation of step counts. However, as all patients in our study were monitored using the same device, systematic measurement bias is unlikely to have affected the between-group comparison. These aspects should be considered when interpreting absolute step counts, although relative differences between groups remain informative.

Baseline mobility was not specifically assessed prior to the intervention. Although early postoperative step counts (POD 0) were recorded, differences in individual functional status may have influenced postoperative activity patterns and could represent a potential confounding factor when interpreting between-group differences.

In essence, our study suggests that a smart wearable device combined with structured reminders increased postoperative mobilization within the first five days after surgery. This protocol is simple to implement, requires no additional staff resources, and may encourage patients to overcome initial hesitation to ambulate. From a perioperative care perspective, these findings support the integration of automated mobilization prompts into standardized recovery pathways, particularly in minimally invasive colorectal surgery where physiological reserve may allow patients to engage more effectively in early activity. Future studies should explore individualized motivational strategies and assess whether enhanced mobilization translates into improved clinical recovery outcomes.

## Conclusion

Smart wearables are simple and secure to use for monitoring postoperative mobility among patients who have undergone elective colorectal resections. The use of these techniques, combined with an hourly alarm signal, was associated with increased patient step counts within five postoperative days.

## Supplementary Information

Supplementary Material 1.

Supplementary Material 2. Table S1. Daily step counts (POD 0–5) (median [IQR]); P values from between-group comparisons. Table S2. Subjective feedback questionnaire.

## Data Availability

The datasets generated and analyzed during the current study are available from the corresponding author on reasonable request.
