# Supplementary material for: Improvement of mobility and motivation in patients with elective colorectal resection using tracking devices and utilizing self-awareness (IMPETUS): a randomized controlled trial in a traditional non-ERAS clinical setting
Source: BMC Surg. 2026 May 27;26:356. doi: 10.1186/s12893-026-03864-6 (PMC13214435; doi:10.1186/s12893-026-03864-6)
Supplement: Supplementary file 2 — Supplementary Material 2. Table S1. Daily step counts (POD 0–5) (median [IQR]); P values from between-group comparisons. Table S2. Subjective feedback questionnaire. [file 12893_2026_3864_MOESM2_ESM.docx]

**Supplementary**

**Table S1 Daily step counts (POD 0–5)** (median [IQR]); P values from between-group comparisons.

| **Postoperative day** | **Intervention group** | **Control group** | **p-value** |
| --- | --- | --- | --- |
| POD 0 | 81 [0–226] | 13 [0–36] | 0.088 |
| POD 1 | 473 [122–950] | 92 [20–226] | **0.013** |
| POD 2 | 790 [51–1418] | 153 [72–482] | 0.155 |
| POD 3 | 1385 [109–3068] | 278 [156–1382] | 0.106 |
| POD 4 | 1553 [306–4298] | 237 [135–1981] | **0.033** |
| POD 5 | 1787 [647–4182] | 449 [153–2148] | 0.083 |

**Table S2** Subjective feedback questionnaire.

| **Question** | **Group A** | **Group B** | **p value** |
| --- | --- | --- | --- |
| Did using the smart wearable physically or mentally stress you? | 1 (1;1) | 1 (1;1) | 0.80 |
| Did you find the technical use of the smart wearable difficult? | 2 (1;2) | 3 (1;2) | 0.98 |
| How much do you think wearing the smart wearable affected the course of your illness? | 3 (3;4) | 3 (2;4) | 0.24 |
| Do you feel more motivated to be active by using the smart wearable? | 4 (3;4) | 3 (2;3.25) | **0.016** |
| Do you think you have had any personal benefit from wearing the smart wearable? | 3 (2;4) | 3 (2;4) | 0.68 |

1 = strongly disagree, 2 = disagree, 3 = neutral, 4 = agree, 5 = strongly agree.

Data are reported as median an interquartile range.
